# Cloning of *CgWRKY53* from *Cymbidium goeringii* and Functional Analysis of Its Negative Regulatory Role in Response to Cold Stress

**DOI:** 10.3390/genes17040376

**Published:** 2026-03-26

**Authors:** Dongrui Ma, Xijun Jing, Lianping Wang, Fengrong Hu

**Affiliations:** 1 College of Life Sciences, Nanjing Normal University, Nanjing 210023, China; 2 College of Landscape Architecture, Nanjing Forestry University, Nanjing 210037, China

**Keywords:** *Cymbidium goeringii*, WRKY genes, low-temperature stress, transgenic

## Abstract

**Background:** *Cymbidium goeringii*, one of China’s traditional and valuable orchids, possesses significant ornamental and economic value. However, it is relatively sensitive to low temperature and other abiotic stresses, which severely restrict its application in landscaping and industrial development. WRKY transcription factors play important roles in plant responses to abiotic stresses, yet related research in *C. goeringii* remains limited. **Methods:** In this study, based on transcriptome data of *C. goeringii* under four different stresses, we identified and cloned the WRKY transcription factor gene *CgWRKY53*. Through bioinformatics analysis, quantitative real-time PCR, and heterologous transformation in *Arabidopsis thaliana*, we systematically investigated its structural characteristics, expression patterns, and function under cold stress. **Results:** The full-length CDS of *CgWRKY53* is 1080 bp, encoding a protein of 359 amino acids with a molecular weight of 39.95 kDa. Group III subfamily of the WRKY family, possessing the conserved WRKYGQK domain and a C2HC-type zinc finger motif. *CgWRKY53* is expressed in roots, pseudobulbs, leaves, and flowers of *C. goeringii*, with the highest expression observed in flowers. Under cold, heat, waterlogging, and ABA treatments, *CgWRKY53* displayed significant changes in expression, with the most pronounced response occurring under cold stress, where its expression was significantly upregulated. Homozygous transgenic *A. thaliana* lines overexpressing *CgWRKY53* exhibited dwarfed stature, with smaller and deformed leaves and notably shorter roots compared to wild-type plants. The overexpression lines also showed cold-sensitive phenotypes under low-temperature stress, and the expression of several cold-responsive genes was suppressed, suggesting that *CgWRKY53* may act as a negative regulator in the response to cold stress. **Conclusions:** These results identify *CgWRKY53* as a negative regulator of cold stress response in *C. goeringii*. This study provides important genetic resources and theoretical foundations for molecular breeding of stress-resistant orchids.

## 1. Introduction

WRKY transcription factors (TFs) participate in multiple processes, including plant hormone signal transduction and MPK signalling cascades, playing a widespread role in plant growth and development, as well as stress response processes [1]. The WRKY family exhibits distinctive structural features, characterised by a highly conserved N-terminal domain—the WRKYGQK heptapeptide sequence. Its C-terminal region additionally contains a metal-chelating zinc finger structure, classified as either the C2H2 type (C-X4-5-C-X22-23-HXH) or the C2-HC type (C-X7-C-X23-HXC) [2]. WRKY transcription factors specifically bind to W-box cis-elements via the conserved WRKYGQK heptapeptide and zinc finger domains, directly regulating cold response genes such as *COR*, *CBF*, and *RD29A*. They are confirmed as core switches in cold signalling networks in model plants like *A. thaliana* and *Oryza sativa* [3]. Based on the number of WRKY domains and the type of zinc finger structure, WRKY transcription factor family members can be classified into three subfamilies: Group I, Group II, and Group III. Group I contains two WRKY domains and one C2H2-type zinc finger structure; Group II contains one WRKY domain and one C2H2-type zinc finger structure ending with an HXC motif; Group III contains one WRKY domain and one C2HC-type zinc finger structure. In addition, Group II is divided into five subgroups, IIa, IIb, IIc, IId, and IIe, based on sequence similarity [4]. Group III contains only one WRKY domain and is characterised by a C2HC zinc finger. Previous studies have demonstrated that Group III members can finely regulate plant stress adaptation through multiple mechanisms, including modulating the ABA signalling pathway, influencing antioxidant systems, or participating in biotic stress responses. The transcription factors *IgWRKY50* and *IgWRKY32*, isolated from *Iris germanica*, act as positive regulators of the ABA signalling pathway. They significantly enhance drought tolerance in transgenic *Arabidopsis* and partially improve its antioxidant system [5]. In *O. sativa* subsp. *indica*, *OsWRKY45* forms a transcription activation complex by interacting with the Mediator subunit OsMED16, recruiting RNA polymerase II to synergistically activate the expression of diterpene phytotoxin synthesis genes, thereby positively regulating rice blast disease resistance [6]. The *OsWRKY71* transcription factor isolated from *O. sativa* positively regulates cold responses. Its expression is rapidly induced by cold stress, and its heterologous expression enhances antioxidant enzyme activity and upregulates key genes in the *CBF* pathway in *A. thaliana*, thereby improving plant cold tolerance [7]. The *GhWRKY21* transcription factor cloned from *Gossypium hirsutum* negatively mediates drought stress responses by finely regulating the expression of GhHAB, a key gene in the ABA signalling pathway. It significantly enhances cotton’s drought tolerance and its capacity for osmotic regulation [8].

However, research on the WRKY family in orchids remains largely unexplored: only *DoWRKY2* has been cloned, and its cold response function remains uncharacterized. There is no evidence regarding the presence of Group III members in *C. goeringii*, their regulatory direction (activation or repression), or their cross-talk mechanisms with ABA signaling. *Song Mei*, belonging to the *C. goeringii* category, is a perennial herb with a cultivation history spanning over two centuries. Flowers bloom singly at the stem apex, featuring three outer petals that are rounded and compact, resembling plum blossoms. The petals have a thick texture, with thin, slightly curled, inward-tucked edges, predominantly displaying a light green base colour. Under varying light conditions, the flower colour may change to deep green, emerald green, or yellowish-green [9]. This study utilized the cold-sensitive cultivar *Song Mei* as material. Based on 4 °C transcriptome data, the significantly differentially expressed Group III subfamily gene *CgWRKY53* was identified. Through cloning, expression profiling analysis, heterologous expression in *A. thaliana*, and low-temperature phenotype characterization, the functional differentiation of this gene was systematically elucidated.

## 2. Materials and Methods

### 2.1. Plant Materials and Treatments

The *Song Mei* plants used in this experiment were obtained from the Institute of Horticulture, Zhejiang Academy of Agricultural Sciences (Hangzhou, China). They were cultivated in well-aerated, water-retentive humus, under natural light conditions, at a temperature of 23 °C and a humidity of approximately 75%, with good ventilation. The planting containers were about 30 cm in height. For tissue-specific expression analysis, healthy two-year-old *Song Mei* plants were selected, and samples were collected from roots, pseudobulbs, mature leaves, flowers, and different floral organs (sepals, petals, labellum, and column). For each tissue type, samples from three individual plants were collected as biological replicates. All samples were immediately frozen in liquid nitrogen after collection and stored at −80 °C for later use. For stress-induced expression analysis, several pots of robust, disease- and pest-free, two-year-old plants (three plants per pot) were selected and subjected to four types of abiotic stress treatments: low temperature (4 °C), high temperature (42 °C), waterlogging, and ABA treatment. For ABA treatment, plants were sprayed uniformly with 100 μM abscisic acid (ABA) solution (prepared by dissolving 264.32 μg ABA powder in 1000 mL water, followed by filtration sterilization) until runoff. Control plants were not treated with ABA. For all treatments, 50 mg of young leaves were sampled at 0, 2, 6, 12, 24, and 48 h post-treatment and used for subsequent RNA extraction and expression analysis. A total of three biological replicates were established for each treatment, with each replicate consisting of pooled leaf samples from three individual plants.

All transgenic plants used in this study were wild-type (WT) *A. thaliana* grown in a growth chamber at 23 °C, with a light intensity of 8000 Lx, 70% humidity, 16 h of light, and 8 h of darkness. The relative humidity in the chamber was maintained at 70%, and the temperature at 23 °C. After approximately 35 days of growth, siliques and inflorescences were removed to prepare for genetic transformation. T_3_ generation transgenic overexpression plants were subjected to cold treatment at 4 °C; at various time points during the stress treatment, samples from three plants were collected, cut into 2 cm segments, and stored in an ultra-low-temperature freezer.

### 2.2. WRKY Gene Clone

WRKY gene sequences were retrieved from the PlantTFDB database. Using leaves from the *Song Mei* spring orchid as material, total RNA was extracted using the kit was purchased from Vazyme, Nanjing, China. The first-strand cDNA was synthesised in a 20 µL system using reverse transcriptase. To prepare for PCR, the cDNA solution was diluted to a concentration that ensured optimal amplification efficiency. Using the *CgWRKY* gene sequence, specific primers for *CgWRKY53* were designed with Oligo6 (Table 1). These primers were employed for high-fidelity amplification and cloning of the full-length CgWRKY gene under the following conditions: 94 °C for 3 min; 32 cycles (98 °C for 10 s, 56 °C for 15 s, 72 °C for 30 s); followed by 5 min at 72 °C. Following amplification, the PCR products were resolved on a 1.5% agarose gel and subsequently purified. The purified fragment was subcloned into the pEASY-blunt vector and transformed into *Escherichia coli* for propagation. Following sequence verification of positive clones, the full-length *CgWRKY53* coding region was obtained and employed as the foundation for subsequent genetic construct development.

### 2.3. Preparation of the PBI121-CgWRKY53 Vector and Its Introduction into Arabidopsis

The PBI121 plasmid containing the 35S promoter was subjected to double digestion with XbaI/SmaI, XbaI/SnaBI, and SmaI/SnaBI. The CgWRKYs fragment and the linearised PBI121 fragment were recovered separately. Following ligation and transformation, the correct strains were selected for testing. The correctly sequenced PBI121-CgWRKYs construct was verified by restriction enzyme digestion. To generate transgenic lines *A. thaliana* plants were transformed via the floral dip procedure. Following seed collection, transformants were identified by germinating the seeds on selective MS medium. After 24 h of dark incubation, the resistance of the screened plants was evaluated until T_3_ transgenic plants were obtained.

### 2.4. Low-Temperature Treatment of T3-Generation Transgenic Material

WT Col-0 and T3-generation transgenic inbred lines were cultured for 35 days in an artificial climate chamber at 23 °C with a light intensity of 8000 Lx. Plants were transferred to a low-temperature incubator maintained at 4 °C under dark conditions and 70% humidity for cold stress. Leaf samples were collected at 0 h, 2 h, 6 h, 12 h, 24 h, and 48 h post-transplant. Following excision, plant tissues were immediately frozen in liquid nitrogen and kept at −80 °C until use. Light and low-temperature signaling pathways interact; light exposure can influence cold response gene expression, potentially confounding the assessment of cold-specific responses [10]. To eliminate this interference and accurately evaluate the response of CgWRKY53 to cold stress, we performed low-temperature treatments under dark conditions [11]. For ABA treatment, *C. goeringii* plants were sprayed with 100 μM ABA (in 0.1% ethanol) or solvent control; leaves were collected at 0–48 h (three replicates). In *Arabidopsis* assays, seeds were germinated on 0–2 μM ABA medium (germination at 7 d), and 4-d-old seedlings were transferred to 0 or 2 μM ABA medium for root measurement at 10 d. All experiments had three biological replicates.

### 2.5. Analysis by Real-Time Quantitative PCR

RT-qPCR experiments were performed using TB Green Premix Ex Taq (Takara, Kusatsu, Japan) on a qRT-PCR instrument, and each qRT-PCR reaction was carried out in a final volume of 10 µL. The reaction mixture consisted of 5 µL of 2× TB Green Premix Ex Taq, 0.2 µL of 50× ROX Reference Dye, 0.2 µL of each gene-specific primer (forward and reverse), 1 µL of diluted cDNA, and 3.4 µL of nuclease-free ddH_2_O. Thermal cycling conditions were set as follows: an initial denaturation at 95 °C for 3 min, followed by 40 amplification cycles comprising 95 °C for 3 s and 60 °C for 30 s. The *C. goeringii* 18S rRNA gene served as the endogenous reference for normalization. Relative expression levels were calculated using the 2^−ΔΔCt^ method. Each experiment was performed with three independent biological replicates, each including three technical replicates. All primer sequences employed for qRT-PCR are provided in Table 1.

### 2.6. Data Analysis

On the NCBI online platform, sequence alignment of CgWRKY genes was performed using Blastx or Blastp (https://blast.ncbi.nlm.nih.gov/Blast.cgi, accessed on 4 February 2026) to analyze whether the sequences contained complete open reading frames and conserved domains. The online tool ProtParam (https://web.expasy.org/protparam/, accessed on 2 February 2026) was employed to predict biochemical characteristics of the WRKY protein from *C. goeringii*, which encompassed the protein’s molecular mass and isoelectric point, instability coefficient, hydrophilicity coefficient, and amino acid composition. Secondary structure prediction of CgWRKY proteins was performed using SOPMA online website (https://npsa.lyon.inserm.fr/cgi-bin/npsa_automat.pl?page=/NPSA/npsa_sopma.html, accessed on 1 February 2026) Subcellular localization prediction was conducted via the WOLF PSORT online tool (http://www.genscript.com/psort/wolf_psort.html, accessed on 1 February 2026), while conserved domain prediction utilized SMART (http://smart.emblheidelberg.de/, accessed on 27 January 2026). WebLogo 3 (for constructing sequence tags) was employed for online analysis. Homologous sequence alignment and phylogenetic analysis were performed using DNAMAN9.0 and MEGA7.0 software, respectively. The amino acid sequences for AtWRKY and OsWRKY proteins were retrieved from the TAIR9.0 database (http://www.Arabidopsis.org/index.jsp, accessed on 4 February 2026) and the Plant Transcription Factor Database (http://planttfdb.cbi.pku.edu.cn/, accessed on 1 February 2026). Sequences of WRKY proteins from Arabidopsis, rice, and tobacco within the WRKY gene family were categorized. Using MEGA 7.0 software and the NJ method with a Bootstrap value of 1000, phylogenetic trees were constructed for CgWRKYs, AtWRKYs, OsWRKYs, and NtWRKYs [12]. Using Kinase Phos (http://kinasephos.mbc.nctu.edu.tw/, accessed on 1 February 2026) revealed that the protein encoded.

## 3. Results

### 3.1. Molecular Cloning and Structural Characterization of CgWRKY53 in C. goeringii

**Figure 1:**
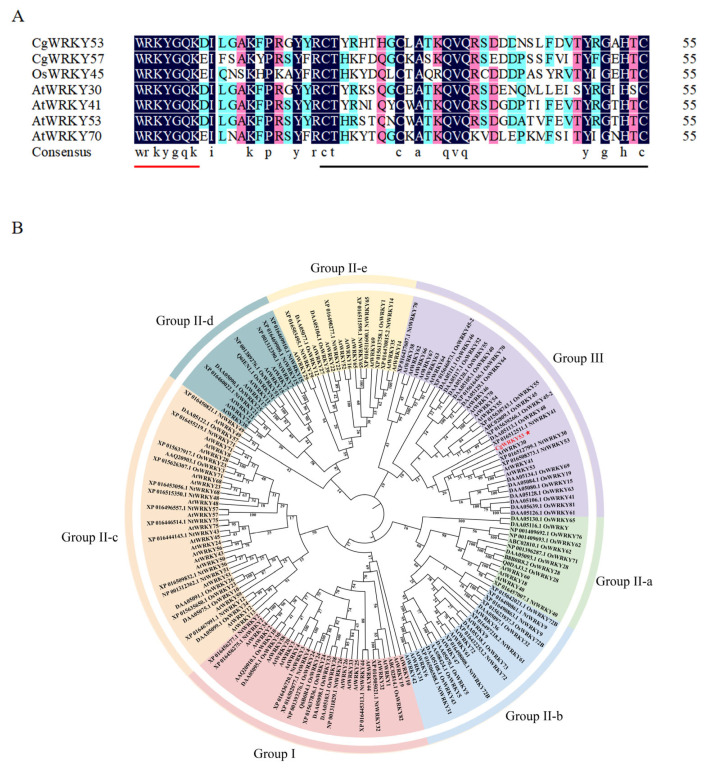
Evolutionary analysis of WRKY domains in *C. goeringii*, *O. sativa*, and *A. thaliana* using sequence alignment and phylogenetic tree construction. (**A**) Alignment of conserved WRKY domain amino acid sequences from *C. goeringii*, *O. sativa*, and *A. thaliana.* Red line: Marks the highly conserved WRKYGQK heptapeptide, the hallmark motif of the WRKY transcription factor family. Black line: Indicates the conserved cysteine (C) and histidine (H) residues of the C2HC-type zinc finger motif, a defining feature of the Group III subfamily; (**B**) Phylogenetic analysis of CgWRKY53. The evolutionary tree was generated using MEGA 7.0 software based on the neighbor-joining (NJ) algorithm with 1000 bootstrap replicates. Based on topological structure, the tree was partitioned into seven distinct clades, designated as Groups I, IIA–E, and III. Note: *C. goeringii*: CgWRKYs; *A. thaliana*: AtWRKYs; *O. sativa*: OsWRKYs; *Nicotiana tabacum*: NtWRKYs, XP: WRKY registration number of *N. tabacum*.

Based on the differential expression profile of the 4 °C low-temperature transcriptome, this study identified a WRKY unigene that was significantly upregulated. Prediction of its open reading frame (ORF) and annotation of conserved domains both classified it as belonging to the Group III subfamily. Using cDNA from young leaves of the *Song Mei* plum cultivar as a template, high-fidelity PCR amplification was performed with gene-specific primers. Agarose gel electrophoresis revealed a single band corresponding to the expected size. The target fragment was cloned into the pEASY-Blunt vector and transformed into *E. coli.* Positive clones were selected for bidirectional sequencing. Results revealed that the *CgWRKY53* open reading frame spans 1080 bp and is 100% identical to the original transcriptome contig. Using Blastp and DNAMAN software, we performed amino acid homology comparisons between the known CgWRKY57 from *C. goeringii*, the CgWRKY53 protein from *C. goeringii*, and WRKY proteins from *Arabidopsis* and rice (Figure 1A). In line with previous classification criteria for WRKY proteins, analysis confirmed that the *CgWRKY53* gene product exhibits the conserved WRKYGQK domain as well as a zinc finger structure characteristic of the WRKY family, which together place it unequivocally within the Group III subfamily.

To further investigate the phylogenetic evolution of CgWRKY proteins and their relationships with other species, WRKY gene sequences from *Arabidopsis*, rice, and tobacco were retrieved from the PlantTFDB database. The WRKY sequences were categorized, and a phylogenetic tree was constructed for CgWRKYs, AtWRKYs, OsWRKYs, and NtWRKYs using MEGA 7.0 (Figure 1B), with 1000 bootstrap replicates. The results showed that WRKY proteins belonging to the same group clustered together. CgWRKY53 clustered with Group III WRKY proteins, exhibiting the closest phylogenetic relationship to AtWRKY53 and AtWRKY70. Beyond illustrating evolutionary relationships among family members, the phylogenetic tree also offered indirect evidence for potential functional conservation. Members of the WRKY family exhibiting analogous functions tended to occupy neighboring branches. For example, AtWRKY50 and AtWRKY51 in Group IIc clustered on the same branch, both playing synergistic roles in inhibiting seed germination and resisting oxidative stress [13]; AtWRKY33 and AtWRKY25 are phylogenetically closest on the tree, both inducing camalexin synthesis during resistance to *Botrytis cinerea* and maintaining high colinearity due to selective pressure [14]. This clustering pattern further suggests that CgWRKY53 may play a similar role in plant stress responses or developmental regulation.

After obtaining the full-length sequence of CgWRKY53, its encoded product was systematically characterized using online tools and local software. When predicting protein hydrophobicity, a negative total average hydrophobicity value indicates a hydrophilic protein, with greater negative values corresponding to stronger hydrophilicity [15]. Proteins with an instability index below 40 were predicted as stable, while those above 40 were predicted as unstable [16]. Physicochemical analysis indicates that CgWRKY53 has an instability index below 40, confirming its stability. Based on the transmembrane helix region prediction results for CgWRKY proteins (Figure S1A), CgWRKY53 is entirely located outside the membrane with no transmembrane domains. Subcellular localization prediction analysis using the WOLF PSORT online tool (Table 1) indicates the highest predicted value (8.40) for nuclear localization of CgWRKY53, suggesting the protein may ultimately localize to the nucleus. Protein phosphorylation site prediction using Kinase Phos revealed that the protein encoded by the CgWRKY53 gene contains 14 serine, threonine, and tyrosine phosphorylation sites. Serine phosphorylation sites were the most abundant in CgWRKY proteins, with CgWRKY53 possessing seven serine phosphorylation sites. This suggests that CgWRKY53 protein activity may be regulated by phosphorylation. SignalP 4.1 analysis of the CgWRKY53 protein from *C. goeringii* revealed no signal peptide (Figure S1B), indicating that CgWRKY53 is not a secreted protein. Secondary structure prediction of CgWRKY proteins using SOPMA software (Figure S1C) revealed that CgWRKY53 comprises 61.00% random coil, 24.79% α-helix, 9.75% extended chain, and 4.46% β-sheet structures.

### 3.2. Tissue-Specific Expression of CgWRKY53 Within Specific Tissues and Following Abiotic

#### Stress Conditions

**Figure 2:**
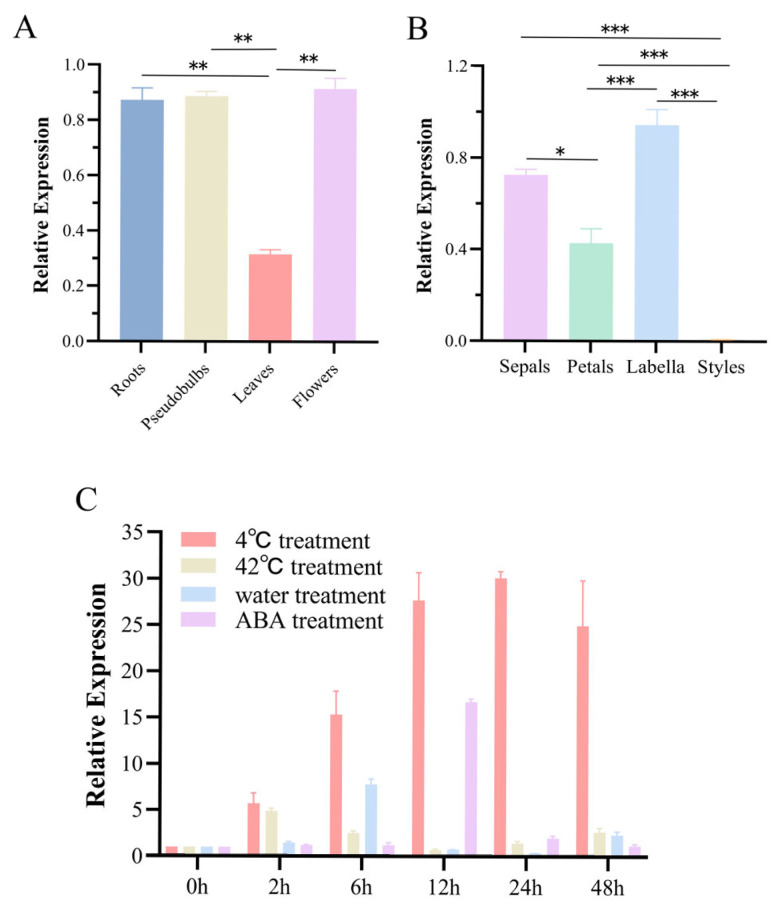
Expression Analysis of *CgWRKY53* in different tissues, organs, and under stress conditions. (**A**) Expression patterns of *CgWRKY53* in different tissues; (**B**) Expression patterns of *CgWRKY53* in different floral organs; (**C**) Expression patterns of *CgWRKY53* under 4 °C, 42 °C, water immersion, and ABA treatments. Data are means ± SD of three biological replicates. (*: *p* < 0.05; **: *p* < 0.01; ***: *p* < 0.001).

To explore the possible functions of *CgWRKY53* across four distinct tissue types, cDNA templates were selected from *C. goeringii* roots, pseudobulbs, leaves, and flowers. Following reverse transcription, quantitative analysis of *CgWRKY53* gene expression was performed using qRT-PCR. Expression levels of this gene were also detected in different floral organs (sepals, petals, labella, and styles) of *C. goeringii*. Results revealed that *CgWRKY53* exhibited the highest relative expression in flowers and pseudobulbs, moderate expression in roots, and significantly lower expression in leaves, demonstrating distinct tissue specificity (Figure 2A). As an energy storage and water-regulation organ in orchids, the marked accumulation of *CgWRKY53* transcripts within pseudobulbs points to a possible function in energy metabolism and stress adaptation. Analysis of expression levels across floral organs revealed that *CgWRKY53* expression in the labellum was approximately 1.7-fold higher than in sepals, 2.1-fold higher than in petals, and 3.5-fold higher than in the gynostemium, thus exhibiting a significantly higher relative expression level in the labellum compared to the sepals, petals, and column (*p* < 0.05), forming a gradient distribution of “labellum > sepal > petal > column” (Figure 2B).

To investigate the differential expression of CgWRKY under conditions of chilling (4 °C), heat stress (42 °C), root waterlogging, and ABA supplementation, we selected cDNA from *C. goeringii* leaves as templates. Using the expression level at 0 h as the control, we first subjected the orchid to low-temperature treatment at 4 °C to analyze the gene’s response to cold stress. Results indicated that under cold stress, the relative expression of *CgWRKY53* progressively increased, peaking at 24 h post-treatment at 30.12-fold relative to the control. At 48 h, relative expression slightly decreased but remained elevated compared to the control. This suggests cold positively regulates *CgWRKY53* expression in leaves. Following exposure of leaves to 42 °C high-temperature stress, *CgWRKY53* expression rapidly increased at the onset of stress, peaking at 2 h post-treatment with a relative expression level 4.90 times higher than the control. Expression decreased at 12 h, then gradually increased thereafter, exhibiting an overall expression pattern of “upregulation–downregulation–upregulation.”

**Figure 3:**
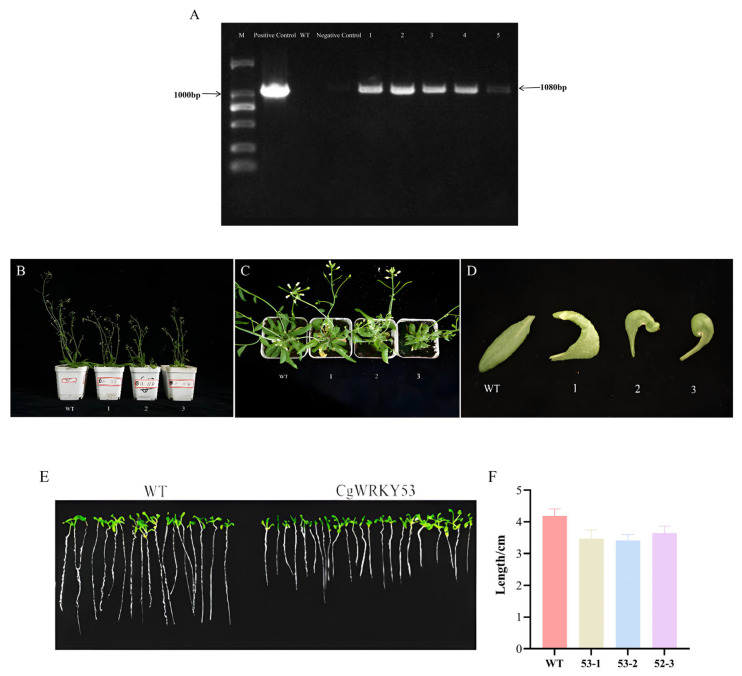
PCR confirmation and analysis of morphological traits in T3 transgenic plants overexpressing *CgWRKY53*. (**A**) PCR detection of CgWRKY53 in transgenic lines overexpressing the gene; (**B**) Side profile of *CgWRKY53*-overexpressing transgenic individuals and white transgenic plants; (**C**) Aerial view of *CgWRKY53*-overexpressing transgenic lines and white genetically modified plants; (**D**) Leaves of *CgWRKY53*-overexpressing transgenic plants; (**E**,**F**) Root length measurements of *CgWRKY53*-overexpressing transgenic plants. Note: WT denotes WT *Arabidopsis*.

**Figure 4:**
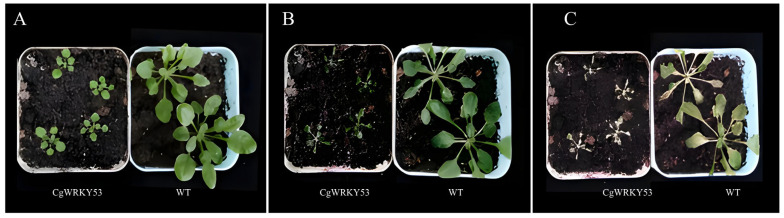
Effects of low-temperature stress on transgenic *Arabidopsis* phenotypes. (**A**) Phenotype after 0 days of low-temperature treatment; (**B**) Phenotype after 6 days of low-temperature treatment; (**C**) Phenotype after 10 days of low-temperature treatment. Note: WT denotes wild-type *Arabidopsis*.

**Figure 5:**
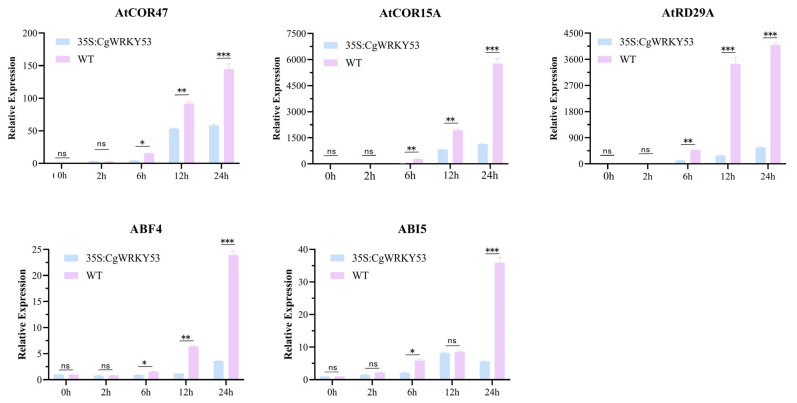
Analysis of *AtCOR47*, *AtCOR15A*, *AtRD29A*, *ABF4*, and *ABI5* gene expression in WT and *CgWRKY53* transgenic plants under low-temperature stress. Data are means ± SD of three biological replicates (*: *p * < 0.05; **: *p* < 0.01; ***: *p* < 0.001; ns: not significant).

To simulate waterlogging stress, the roots were placed in standing water. The relative expression changes in the gene are shown in Figure 3, Figure 4 and Figure 5. Compared to the control, the relative expression of *CgWRKY53* showed no significant difference at 2 h post-treatment. It rapidly peaked at 6 h, reaching 7.79 times the control level, followed by downregulation. Relative expression then gradually increased again at 48 h, exhibiting an overall expression pattern of “upregulation–downregulation–upregulation.”

Plant hormones such as ABA play crucial roles in regulating plant growth and development and in signaling networks that respond to biotic and abiotic stresses. This study applied 100 μM ABA to leaves via overhead spraying until dripping to assess how WRKY genes respond to externally applied ABA. The relative transcript level of *CgWRKY53* showed no significant trend in the early treatment phase (<6 h). Relative expression peaked at 12 h post-treatment, reaching 16.71-fold higher than the control, with no significant trend thereafter (Figure 2C). These results indicate that *CgWRKY53* exhibits a significant response to low-temperature stress and a strong response to ABA stress. We acknowledge that qRT-PCR data were normalized to 18S rRNA; while this is a commonly used reference gene in orchid studies, we recognize that stress-induced fluctuations may introduce variability. We sincerely urge readers to interpret these findings with this limitation in mind.

### 3.3. Identification and Phenotypic Observation of Transgenic Plants with Overexpression

PCR detection was performed using the 35S forward primer and *CgWRKY53* reverse primer, with DNA from T_3_ transgenic and WT plants, as well as the PBI121-CgWRKY53 plasmid and water as templates. Results showed that the target product was not amplified from WT DNA, whereas the target product was readily obtained from T3 transgenic genomic DNA. These results confirm the successful integration of *CgWRKY53* into the wild-type genome (Figure 3A). At seven days post-germination, WT seedlings were transferred to soil-filled pots and maintained in a controlled-environment growth chamber. Phenotypic evaluation of transgenic lines was performed after an additional 30 days of cultivation. Compared with WT, plants overexpressing *CgWRKY53* exhibited delayed growth and development, dwarfism, reduced overall leaf size, and curled, deformed leaf shape (Figure 3B). Among these, Line 1 exhibited minor leaf deformation, Line 2 showed partial leaf reduction with severe deformation, and Line 3 demonstrated overall leaf reduction (Figure 3D). Additionally, transgenic plants exhibited premature stem elongation and flowering, indicating that *CgWRKY53* suppressed vegetative growth. Seeds harvested from *CgWRKY53*-overexpressing lines and WT plants were plated on MS solid medium to initiate germination for subsequent root length analysis, with root length recorded after 14 days. Results showed that both transgenic and WT roots exceeded 30 mm in length. However, the root elongation in the *CgWRKY53* transgenic homozygous line was significantly shorter than that of WT, with no significant difference in root morphology between the two (Figure 3E,F). This indicates that *CgWRKY53* inhibits both shoot vegetative growth and root development in plants.

### 3.4. Expression Analysis of CgWRKY53 Transgenic Plants Under Low-Temperature Stress

To further investigate the role of *CgWRKY53* in cold stress regulation, transgenic CgWRKY and WT *Arabidopsis* cultured for approximately 21 days were subjected to 4 °C cold stress. After 6 days of 4 °C treatment, all treated plants exhibited varying degrees of wilting. Compared with WT, leaves of *CgWRKY53* Transgenic plants exhibited slight curling. After 10 days at 4 °C, the leaves of both *CgWRKY53*-transgenic and WT plants turned yellow and dried, with partial plant death observed. Collectively, these results indicate that *CgWRKY53*-transgenic plants exhibit lower cold tolerance than WT (Figure 4A–C). Although quantitative physiological metrics (electrolyte leakage, MDA content, ROS accumulation, Fv/Fm) were not assessed in this study, the suppression of cold-responsive marker genes (*AtCOR47*, *AtCOR15A*, *AtRD29A*) and ABA signaling components (*ABF4*, *ABI5*) provides molecular-level evidence supporting the negative regulatory role of *CgWRKY53*. This finding is consistent with recent advances in WRKY functional studies. In *Anoectochilus roxburghii* (Orchidaceae), ArWRKY57 and ArWRKY70—both Group III WRKY proteins phylogenetically related to CgWRKY53—form a heterocomplex and directly regulate *ArLEA5* transcription by binding to W-box elements in its promoter, as validated by yeast two-hybrid (Y2H), bimolecular fluorescence complementation (BiFC), and yeast one-hybrid (Y1H) assays [17]. This provides the first direct evidence of WRKY-mediated transcriptional regulation in orchids. Furthermore, studies on AtWRKY53, the closest *Arabidopsis* homolog of CgWRKY53, have revealed that WRKY53 forms protein complexes with antioxidant enzymes, creating a feedback regulatory loop that fine-tunes reactive oxygen species homeostasis [18], and directly binds to the *AtABI3* promoter to regulate ABA signaling [19]. The convergence of these findings from orchids and model plants provides strong indirect support for our conclusion.

To investigate whether the expression levels of stress-related genes associated with cold stress change, real-time quantitative PCR was used to detect downstream gene expression. Although *CgWRKY53* is activated at the transcriptional level under cold stress, its overexpression resulted in significantly lower induction levels of cold response marker genes (*AtCOR47*, *AtCOR15A*, *AtRD29A*) compared to the WT. At the same time, activation of ABA signaling components (*ABF4*, *ABI5*) was also delayed (Figure 5). These results indicate that *CgWRKY53* acts as a negative regulator of cold tolerance, simultaneously suppressing both the ICE–CBF–COR pathway and the ABA-dependent cold signaling pathway. The reduced induction of *AtCOR47*, *AtCOR15A*, and *AtRD29A* in *CgWRKY53*-overexpressing lines provides a molecular explanation for the observed cold-sensitive phenotype, as these genes are well-established effectors of cold acclimation. Furthermore, the expression level of the *ABF4* gene showed no significant difference within 12 h of cold treatment in plants overexpressing *CgWRKY53*, whereas *ABF4* expression accelerated in response to cold stress in the WT, indicating that *CgWRKY53* overexpression slows the speed of *ABF4*’s response to cold stress (Figure 5). This suggests that *CgWRKY53* can reduce plant adaptation to low temperatures by inhibiting downstream cold response pathways.

Collectively, these findings suggest that CgW*RKY53* exhibits strong sensitivity to cold treatment, rendering plants more vulnerable to low temperatures. Overexpression of *CgWRKY53* enhances ABA’s inhibitory effect on seed germination and root development while exacerbating plant damage under low temperatures, suggesting its potential role in regulating plant sensitivity to stress.

## 4. Discussion

*C. goeringii* is a plant appreciated for its decorative characteristics, though its appearance can be negatively affected by exposure to abiotic stressors. Extensive research has established that WRKY transcription factors govern multiple aspects of the plant life cycle, from organogenesis to stress signaling, and members of this family have been characterized in various plant lineages [14]. However, existing reports on *C. goeringii* remain scarce. In this study, we identified a CgWRKY protein. By aligning its conserved WRKY domain with corresponding structures from functionally characterized proteins and constructing a phylogenetic tree, we analyzed its structural characteristics. Analysis results indicate that sequence conservation within the WRKY domain is a universal and prominent attribute of WRKY transcription factors. Many researchers have pointed out that this domain is crucial for the function of CgWRKY genes [20]. The core of this domain is the WRKYGQK sequence, and its variant forms (e.g., WRKYGKK, WSKYEQK, and WRKYSEK) have been identified in multiple species [21]. For instance, WRKYGKK is present in maize, rice, soybean, and *Arabidopsis*, whereas WKKYGQK is present in barley and common bean. WSKYEQK and WRKYSEK have also been found in diverse plant species [22,23]. Although variant forms of the WRKYGQK sequence have been identified across diverse plant species, the core sequence of the CgWRKY domain remains highly conserved, with no mutations, indicating minimal evolutionary changes in its amino acid sequence. Significant differences exist in the zinc finger structures of different WRKY proteins. Researchers classified WRKY transcription factors into three subfamilies based on variations in their zinc finger sequence structures and lengths. Phylogenetic analysis was conducted by constructing a phylogenetic tree. The results indicate that CgWRKY53 clusters with Group III WRKY proteins, showing the closest phylogenetic relationship to AtWRKY53 and AtWRKY70. Thus, CgWRKY53 belongs to Group III, possessing the characteristic C2HC zinc finger structure typical of this group. Similar to SlWRKY52 from Group III in tomato, which enhances drought tolerance by regulating antioxidant enzyme activity and stomatal closure through an ABA-dependent pathway [24]. Although these evolutionary relationships suggest potential functional similarities, phylogenetic proximity alone does not establish functional equivalence; direct experimental evidence, such as the heterologous expression and downstream gene expression analysis provided in this study, is essential for functional characterization.

The WRKY transcription factor family plays a broad and important role in plant growth and development, and gene expression patterns provide important clues for functional studies. WRKY members that are strongly upregulated in specific plant samples often participate in controlling the differentiation and growth of those structures. Therefore, overexpression of WRKY transcription factors frequently leads to diverse growth and development phenotypes in transgenic plants. For example, overexpression of *AtWRKY75* results in earlier flowering and increased plant height, while RNAi-silenced lines exhibit delayed flowering [25]. In this study, we cloned a WRKY gene, designated as *CgWRKY53,* which shows significantly high expression in floral organs. In addition, overexpression of *CmWRKY10* enhances drought tolerance in chrysanthemum, and this mechanism is related to the ABA pathway [26]. However, overexpression of *GhWRKY17* in cotton accelerates leaf senescence and reduces drought tolerance, acting as a repressor of drought tolerance resistance [25]. Ectopic expression of *AtWRKY57* in *Arabidopsis* delays flowering, while its heterologous expression in rice enhances drought tolerance [27]. The same gene, *AtWRKY57*, exhibits dual functions in developmental regulation and stress response in different species. Evidence from these investigations implies that the functional effects of different WRKY transcription factors are significantly differentiated: they can either promote or inhibit specific developmental processes, and the same gene may display pleiotropic phenotypes in different genetic backgrounds. In this study, we cloned a WRKY gene, designated as *CgWRKY53*, which shows significantly high expression in floral organs.

In this research, transgenic *Arabidopsis* overexpressing *CgWRKY53* exhibited a unique combination of phenotypes: plant dwarfism, reduced and curled leaves, premature bolting and flowering, along with significantly shortened root length. This dwarfing trait resembles that of *GhWRKY34* [28], but *CgWRKY53*’s premature flowering contrasts with *GhWRKY34*’s delayed flowering and aligns with *AtWRKY75*’s promotion of flowering [29]. Notably, the negative regulatory effect of *CgWRKY53* on cold tolerance is consistent with the characteristics of *OsWRKY7*. Research indicates that *OsWRKY7*, as a key negative regulator of cold stress response in rice seedlings, exhibits a cold-sensitive phenotype in its overexpression lines, while knockout mutants show significantly enhanced cold tolerance [30]. This finding corroborates the results of this study, in which *CgWRKY53* overexpression increased cold sensitivity in spring orchids, while gene silencing enhanced cold tolerance. This indicates that overexpression of the *CgWRKY53* gene contributes to the positive regulation of plant senescence. As one of the most expansive families of transcriptional regulators in the plant kingdom, WRKY proteins serve as core regulators of integrating multiple environmental signals and coordinating plant responses to stress. Numerous studies have demonstrated that WRKY genes exhibit diverse expression patterns and regulatory mechanisms under various stress conditions. *AtWRKY25* in *Arabidopsis* is rapidly induced by high temperature, reaching peak expression within 2 h before gradually declining [31]; *AtWRKY22* is significantly upregulated after 6 h of waterlogging stress, enhancing water stress resistance by regulating downstream genes such as *MYB15* and *PUB24* [32]; and *CsWRKY2* responds to drought and cold stress in camellia, with its expression significantly increasing when ABA content rises [33]. Furthermore, *DoWRKY5* in Dendrobium catenatum is co-induced by cold stress and ABA, exhibiting time-dependent dynamic expression patterns [34]. These studies indicate that WRKY genes integrate multiple abiotic stress signals to regulate diverse stress responses in plants, yet exhibit significant differences in expression dynamics and functional effects among different genes. Within this work, the expression pattern of *CgWRKY53* following exposure to four stress treatment conditions shares both commonalities and unique features with the aforementioned reports. Under cold stress, *CgWRKY53* peaked at 24 h (30.12-fold induction), resembling the rapid induction pattern of *AtWRKY25* under high temperature, but with a delayed response time. Under high-temperature stress, *CgWRKY53* exhibited an “up-down-up” oscillatory pattern, differing from *AtWRKY25*’s single-peak response; water stress induced significant expression at 6 and 12 h, aligning with *AtWRKY22*’s response timing; and ABA treatment reached a peak at 12 h (16.71-fold), consistent with the ABA response characteristics of *CsWRKY2* and *DoWRKY5*. However, unlike *CsWRKY46*’s positive regulation of cold tolerance [35], *CgWRKY53* overexpression paradoxically reduced plant cold tolerance, exhibiting a “high-expression–low-tolerance” phenotypic contradiction. As mentioned earlier, this negative regulatory pattern is not unique within the WRKY family. In *O. sativa*, *OsWRKY45* acts as a negative regulator, and its overexpression increases sensitivity to low-temperature stress [36,37]. Similarly, *OsWRKY53* negatively regulates rice blast resistance, cold stress tolerance, and salt stress tolerance. Its knockout mutant enhances cold tolerance during the heading stage by suppressing gibberellin content in anthers [38,39]. These studies indicate that members within the same WRKY family or even subfamily may finely regulate the intensity of plant responses to stress through antagonistic mechanisms, and a single WRKY gene may perform diametrically opposed functions under different stresses or developmental stages. In this study, *CgWRKY53* exhibits high expression under low temperatures, yet its overexpression reduces cold tolerance in plants—a pattern highly similar to the negative regulation observed in *AtWRKY70/54*. Phylogenetic analysis indicates *CgWRKY53* shares the closest evolutionary relationship with *AtWRKY53* and *AtWRKY70*. Besseau et al. generated *AtWRKY54*-*AtWRKY70* double mutants by knocking out both *AtWRKY54* and *AtWRKY70*, observing premature leaf senescence in these plants. This finding indicates that both genes participate in the negative regulation of leaf senescence [13], further supporting the possibility that *CgWRKY53* functions as a negative regulator in stress responses. Although direct mechanistic validation—such as electrophoretic mobility shift assay (EMSA) or chromatin immunoprecipitation (ChIP)—was not performed in this study, recent advances provide robust indirect support for DNA binding. The conserved WRKYGQK domain and C2HC zinc finger of CgWRKY53 (Figure 1A) are hallmarks of DNA-binding WRKY proteins. Most importantly, a study in *A. roxburghii* (Orchidaceae) by Jiang et al. (2025) demonstrated that ArWRKY57 and ArWRKY70—both Group III WRKY proteins phylogenetically related to CgWRKY53—directly regulate *ArLEA5* transcription by binding to W-box elements in its promoter, as validated by Y1H assays [17]. This provides the first direct evidence of WRKY-mediated transcriptional regulation in orchids and strongly supports the plausibility that CgWRKY53 operates through a similar DNA-binding mechanism. Additionally, Xu et al. (2026) demonstrated that AtWRKY53 directly binds to the W-box in the promoter of *AtABI3* [19], and the delayed activation of *ABF4* and *ABI5* in our *CgWRKY53*-overexpressing lines (Figure 5) is fully consistent with this established role. While the present study demonstrates that *CgWRKY53* overexpression suppresses cold-responsive gene expression and reduces cold tolerance in *Arabidopsis*, we acknowledge that these conclusions are based solely on heterologous overexpression and that direct DNA-binding evidence is currently lacking. Native validation in *C. goeringii* through virus-induced gene silencing (VIGS) or gene editing is essential to exclude species-specific effects and confirm its endogenous role. Stable transformation of *Cymbidium* species remains technically challenging, with recent advances achieving transformation efficiencies around 18.66% in *C. goeringii* [40]; however, optimization of a VIGS protocol for *C. goeringii* has been prioritized in our future research plan to enable direct functional analysis in the native species. VIGS has been successfully applied in related orchid species such as *Phalaenopsis* for functional gene analysis providing a promising technical reference for future studies in *C. goeringii* [41].

Low-temperature stress is one of the primary environmental factors limiting plant growth, development, and geographic distribution. As a significant abiotic stressor, it induces phase transitions in cell membranes and bursts of reactive oxygen species, leading to osmotic imbalance and disruption of electrochemical gradients. This inhibits photosynthesis and reproductive growth, resulting in severe declines in plant yield and quality [42]. However, WRKY transcription factors exhibit significant functional differentiation, acting both as facilitators that increase cold tolerance and functioning to reduces to limit excessive cold responses. Regarding positive regulation, cucumber *CsWRKY46* is rapidly triggered upon exposure to low temperature, and its ectopic expression dramatically enhances freezing tolerance in engineered *Arabidopsis* lines by activating the expression of cold response genes such as *RD29A* and *COR47* through an ABA-dependent pathway [35]. In wheat, *TaWRKY19* is induced by multiple stresses, and its overexpression significantly enhances the cold tolerance of transgenic Arabidopsis by activating the expression of cold response genes such as *DREB2A*, *RD29A*, *RD29B*, and *Cor6.6*, demonstrating positive regulation against freezing stress [43]. Notably, however, increasing evidence indicates that certain WRKY family members negatively regulate cold response gene expression to prevent plant overreaction. Guo et al. (2024) identified a 60-bp InDel mutation in the *WRKY34* promoter region through comparative genomic analysis of wild and cultivated tomatoes, resulting in significantly reduced *SlWRKY34* expression in cultivated tomatoes. Further validation of *SlWRKY34* function using CRISPR/Cas9 gene editing and overexpression techniques, combined with RNA-seq and ChIP-seq-based downstream target gene screening, ultimately confirmed that *SlWRKY34* negatively regulates tomato cold tolerance by interfering with the *CBF* cold response core pathway at both transcriptional and translational levels [44]. Additionally, Zou et al. (2010) demonstrated, using promoter-GUS fusion and qRT-PCR, that *AtWRKY34* is specifically expressed in mature pollen and is cold-inducible. Functional validation using T-DNA insertion mutants and overexpression lines showed that *AtWRKY34* deficiency enhances pollen cold tolerance, while overexpression induces cold sensitivity and sterility. qRT-PCR analysis confirmed that WRKY34 negatively regulates cold stress responses in mature pollen by blocking the *ICE1-CBF-COR* cold response core pathway through suppression of *CBF* transcription factor and its downstream *COR* gene expression [45]. These studies indicate that high expression of WRKY genes under cold stress does not necessarily equate to positive regulation. Some members may avoid energy expenditure from excessive defense responses by suppressing core cold response pathways or disrupting redox balance. In this study, *CgWRKY53* showed strong induction at 4 °C, resembling the cold response patterns of negative regulators such as *SlWRKY34* and *AtWRKY34*, characterized by rapid early induction. Transgenic Arabidopsis overexpressing *CgWRKY53* showed yellowing and wilting leaves after 10 days of cold treatment, with partial plant death and significantly reduced cold tolerance compared to WT plants. Further downstream gene analysis revealed that *CgWRKY53* overexpression resulted in significantly lower upregulation of key cold signal pathway genes *AtCOR47*, *AtCOR15A*, and *AtRD29A*, and of ABA response genes *ABF4* and *ABI5*, compared to WT plants. Moreover, the response speed of *ABF4* to low temperatures was slowed. Li et al. (2013) constructed single and double mutants of *WRKY54* and *WRKY70*, revealing that the *WRKY54/WRKY70* double mutant exhibited enhanced tolerance to osmotic stress. Further validation through stomatal aperture measurements and water loss rate experiments confirmed that *AtWRKY54* and *AtWRKY70*, both belonging to the WRKY subfamily III, negatively regulate plant tolerance to osmotic stress—including cold-related osmotic stress—by inhibiting stomatal closure [13]. These findings, together with our own data, support a broader view of Group III WRKYs as negative regulators in stress responses. In the present study, we observed a clear distinction between the cold-induced expression of CgWRKY53 and its functional role as a negative regulator: although *CgWRKY53* is transcriptionally activated by low temperature, its overexpression suppresses both the ICE–CBF–COR pathway (as evidenced by reduced COR gene induction) and ABA-dependent cold signaling (as evidenced by delayed *ABF4*/*ABI5* activation). This dual suppression positions *CgWRKY53* as a feedback regulator that fine-tunes cold acclimation by attenuating multiple downstream pathways. This suggests that *CgWRKY53* may likewise function as a Group III subfamily-specific negative regulator, finely modulating the adaptive response intensity of *Song Mei* to low-temperature stress by either directly binding to W-box elements within the regulatory sequences of target cold-responsive genes or forming inhibitory complexes with other transcription factors to block activation of the *ICE1-CBF-COR* pathway.

## 5. Conclusions

This study cloned the CgWRKY53 gene from the spring orchid *Song Mei*, which belongs to subgroup III within the WRKY family transcription factor family. Bioinformatics analysis revealed that the CgWRKY53 protein contains a typical WRKYGQK domain and a C2HC-type zinc finger structure. It exhibits the closest phylogenetic relationship with Arabidopsis AtWRKY53 and AtWRKY70, suggesting potentially similar biological functions. Tissue expression analysis revealed that CgWRKY53 is expressed in all tissues of *C. goeringii*, with the highest expression in flowers, demonstrating distinct tissue specificity. Homozygous T_3_ generation *A. thaliana* lines overexpressing CgWRKY53 were obtained via the Agrobacterium-mediated floral dip transformation method. The phenotypic functions of these lines under various stress conditions were subsequently investigated. The data presented here pave the way for a deeper dissection of the molecular mechanisms by which CgWRKY53 regulates the growth, development, and stress adaptation in *C. goeringii*. Simultaneously, this work provides important genetic resources and theoretical underpinnings for the molecular breeding of stress-resistant orchids.

## Figures and Tables

**Table 1:** Subcellular localization prediction of protein coded by CgWRKY53.

Subcellular Localization	CgWRKY53
Nucleus	8.40
Cytoplasmic membrane	0.82
Extracellular	0.00
Cytoplasm	0.00
Mitochondria	0.06
Golgi apparatus	0.00
Chloroplast	0.17
Vacuole	0.48

## Data Availability

The following information was supplied regarding data availability: Raw data are available in the Supplementary Materials.

## Supplementary Materials

The following supporting information can be downloaded at: https://www.mdpi.com/article/10.3390/genes17040376/s1, Table S1. Primers used for CgWRKY53 gene cloning and qRT-PCR analysis in *C. goeringii*; Figure S1: Bioinformatic analysis of CgWRKY53 protein structure.
