# Supplementary material for: Cloning of CgWRKY53 from Cymbidium goeringii and Functional Analysis of Its Negative Regulatory Role in Response to Cold Stress
Source: Genes (Basel). 2026 Mar 26;17(4):376. doi: 10.3390/genes17040376 (PMC13116840; doi:10.3390/genes17040376)
Supplement: Supplementary file 1 [file genes-17-00376-s001.zip › Supplementary Figures.pdf]

Supplementary Figures

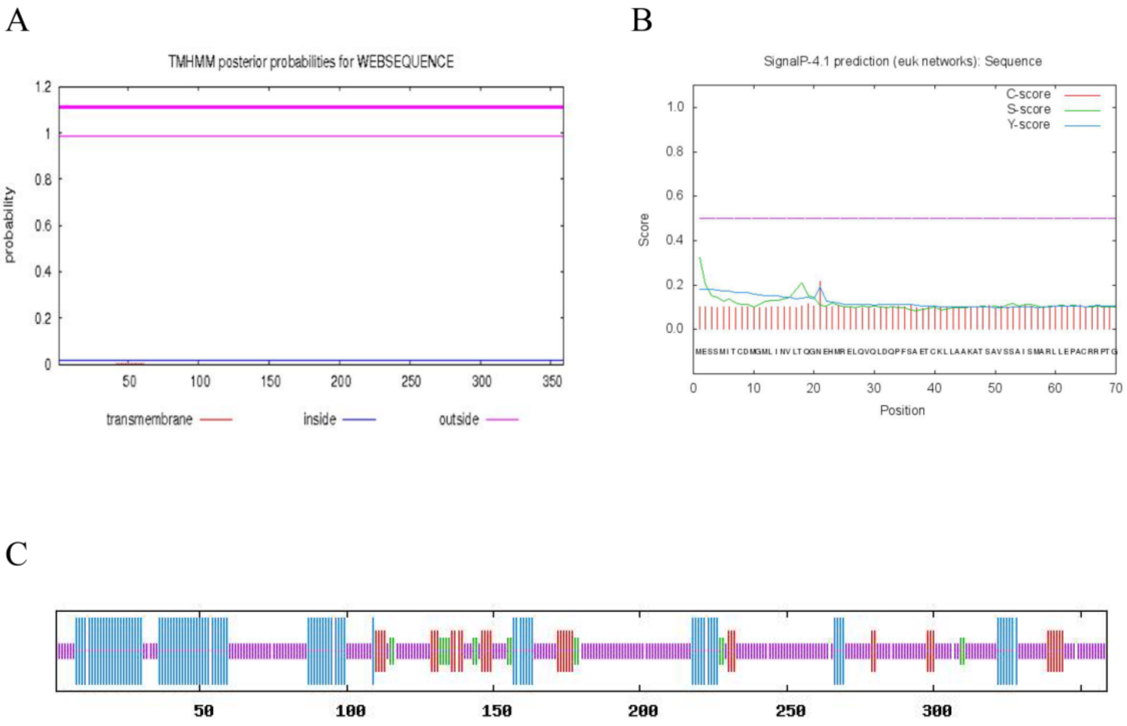

Figure S1: Bioinformatic analysis of CgWRKY53 protein structure.(A) Predicted transmembrane helical structure of CgWRKY53 protein; (B) Predicted signal peptide of CgWRKY53 protein;(C) Secondary structure prediction of CgWRKY53 protein: From left to right, representing the N-terminal to C-terminal regions. Blue indicates α-helix locations, red indicates extended chain locations, green indicates β-sheet locations, orange indicates random coil locations, and pink-purple indicates amino acids without specific structural features.
