# Supplementary material for: Cloning of CgWRKY53 from Cymbidium goeringii and Functional Analysis of Its Negative Regulatory Role in Response to Cold Stress
Source: Genes (Basel). 2026 Mar 26;17(4):376. doi: 10.3390/genes17040376 (PMC13116840; doi:10.3390/genes17040376)
Supplement: Supplementary file 1 [file genes-17-00376-s001.zip › Primers used for CgWRKY53 gene cloning and qRT-PCR analysis in C. goeringii.pdf]

Table S1. Primers used for *CgWRKY53* gene cloning and qRT-PCR analysis in *C. goeringii*.

Primer name	Primer sequence (5' to 3')
CgWRKY53-F	ATGGAGAGCAGCATGATCACCT
CgWRKY53-R	TCAAGATAAACCAAAAGGGAAATC
qCgWRKY53-F	CGACATGGGCATGCTTA
qCgWRKY53-R	CAAGCCGGCTCGAGTAG
18SrRNA-F	GGTCCTATTGTGTTGGCT
18SrRNA-R	TCGCAGTGGTTCGTCTTT
